# Appetite for coping: a latent profile analysis of typological heterogeneity in nurse burnout and emotional eating

**DOI:** 10.3389/fnut.2026.1884663

**Published:** 2026-08-07

**Authors:** Xin Liang, Xiaoru Wu, Hanqing Zhang, Bo Zheng

**Affiliations:** 1 Nanyang First People's Hospital, Nanyang, Henan, China; 2 The First Affiliated Hospital, Zhejiang University School of Medicine, Hangzhou, China; 3 Department of Oncology, Renmin Hospital of Wuhan University, Wuhan, Hubei, China; 4 Faculty of Public Health, Mahidol University, Bangkok, Thailand

**Keywords:** burnout, eating behavior, eating disorders, latent profile analysis, nurse

## Abstract

**Objective:**

To identify the latent heterogeneous subtypes of burnout and emotional eating among Chinese clinical nurses, and to examine the predictive effects of monthly night shift frequency and years of work experience on subtype membership.

**Methods:**

A convenience and snowball sampling method was used to survey 360 practicing nurses in China from August 2025 to January 2026. Burnout and emotional eating/emotional undereating were measured using the Maslach Burnout Inventory-Human Services Survey (MBI-HSS) and the Adult Eating Behavior Questionnaire (AEBQ), respectively. Latent profile analysis (LPA) was conducted to identify subtypes, and multinomial logistic regression was used to examine predictors.

**Results:**

LPA identified three distinct profiles: “Low Burnout-Adaptive Eating” (36.1%), “Moderate Burnout-Emotional Reactive” (47.8%), and “Severe Burnout-Maladaptive Coping” (16.1%). Multinomial logistic regression showed that each one-level increase in monthly night shift frequency was associated with a 72.6% higher risk of belonging to the severe burnout profile (OR = 1.726, *p* = 0.014). Each one-level increase in work experience significantly reduced the odds of being classified into the severe burnout profile by 28.2% (OR = 0.718, *p* = 0.026). Body Mass Index (BMI) was not a significant predictor.

**Conclusion:**

There is a substantial co-occurrence of burnout and emotional eating among Chinese nurses. Frequent night shifts serve as a key driver of resource depletion, while professional experience provides a stepwise protective effect. Nursing administrators should implement refined scheduling to cap monthly night shifts and deploy precision-targeted interventions (e.g., mindful eating support) toward novice nurses with high night shift loads to interrupt early-career loss spirals.

## 1 Introduction

In the 11th Revision of the International Classification of Diseases (ICD-11, 2021), the World Health Organization (WHO) categorizes burnout as a significant health concern (1). Healthcare professionals worldwide are under immense occupational stress, which significantly elevates their risk of burnout—a syndrome characterized by emotional exhaustion, depersonalization, and a diminished sense of personal accomplishment (2, 3). The phenomenon of burnout—defined by profound exhaustion, impaired cognitive and emotional regulation, and psychological detachment—poses a multifaceted threat: it jeopardizes the health and wellbeing of medical staff, generates immense economic burdens, and presents a critical risk to patient outcomes through the potential erosion of care quality (4–6). Official government figures indicate that China's registered nurse population stands at around 5.2 million, which equates to less than 4 nurses per thousand capita. Although the Chinese nursing sector has made significant strides with a rapidly expanding workforce, it still falls short of the World Health Organization (WHO) benchmark of 4.45 nurses per thousand. Consequently, high nurse turnover further intensifies resource scarcity and team instability, ultimately degrading healthcare delivery and elevating risks to patient safety (7, 8). Chinese nurses show an overall burnout prevalence of 64.5 %, with 12.5 % at severe levels (9, 10). Chinese nurses face significant structural pressures that existed long before the COVID-19 pandemic, including high nurse-to-patient ratios, inadequate staffing, frequent night shifts, limited career development opportunities, and a lack of psychological support. These long-standing pressures result in the continuous accumulation of physical and mental burdens among nurses, laying a deep foundation for the onset of occupational burnout (11, 12).

Emotional eating is defined as a non-pathological eating behavior characterized by the tendency to use eating as a strategy to cope with negative emotions. Studies indicate that emotional eating manifests in complex behavioral patterns among individuals, covering both over-eating and under-eating dimensions (13–15). Szweda and Thorne (16) observed disordered eating among certain nursing students presenting with a BMI over 30, underscoring the necessity for further exploration. While existing literature correlates body weight with work efficiency, absenteeism, and eating disorder risks, domain-specific evidence focusing on nurses is still profoundly lacking.

Recent international research has explored the relationship between emotional eating and burnout among nurses. One study of British nurses found that those under high stress tended to consume more snacks, such as chocolate chips and biscuits (17). A survey of 297 nurses in Turkey found that perceived stress was significantly positively correlated with emotional eating, while life satisfaction was negatively correlated with it (18). A study of 168 nurses from a national sample in Thailand indicated that anxiety, depression, and client-related burnout were significant positive predictors of emotional eating (19). Moreover, emotional eating was positively correlated with job performance among nurses, suggesting that this behavior may affect occupational functioning through its impact on personal health (20). In the Chinese nurse population, preliminary research has begun to reveal the influencing factors and underlying mechanisms of emotional eating. A cross-sectional study involving 662 nurses in Hong Kong demonstrated that shift work was significantly positively correlated with emotional eating, and that life satisfaction, perceived stress, and marital status each had predictive effects on nurses' emotional eating levels (20). Furthermore, a study conducted in Mainland China found that emotion regulation difficulties mediated the relationship between burnout and emotional overeating, suggesting that burnout may increase the risk of emotional overeating by impairing nurses' capacity for emotion regulation (21). To date, no study has applied LPA to examine the burnout-emotional eating relationship in the Chinese nursing context.

“The Conservation of Resources (COR) theory (22) posits that individuals strive to acquire, maintain, and protect valued resources, and psychological stress occurs when these resources are threatened or lost. In the demanding clinical environment, nurses must continually invest emotional, cognitive, and physical resources to meet high work demands. When these investments are not adequately replenished, resources become progressively depleted. From a COR perspective, the two core dimensions of burnout—emotional exhaustion and depersonalization—can be conceptualized as an advanced state of resource loss. Emotional exhaustion reflects the depletion of emotional energy reserves, while depersonalization represents a defensive cognitive withdrawal aimed at conserving remaining resources.”

COR theory further proposes that initial resource loss triggers “loss spirals” (23), wherein depleted individuals become increasingly vulnerable to further resource erosion. Difficulties in emotion regulation can be understood as a specific manifestation of this spiral: when emotional resources are exhausted, individuals lose the internal capacity to monitor, evaluate, and modulate negative emotions effectively. In COR terms, emotion regulation constitutes a critical internal regulatory resource; its impairment signals that the loss cycle has extended from energetic depletion into the domain of self-regulatory dysfunction. Without adequate regulatory capacity, nurses are less able to buffer the emotional impact of ongoing workplace stressors, leaving them more susceptible to maladaptive coping responses. (24). To interrupt the loss spiral or compensate for depleted internal resources, individuals may turn to alternative means of resource acquisitional process COR theory describes as resource substitution. Emotional overeating can be viewed as one such substitution strategy: eating palatable food provides rapid sensory pleasure and energy replenishment, temporarily alleviating negative emotional states (25). However, this strategy is inherently maladaptive because it does not address the underlying resource deficit and may create additional health burdens over time.

## 2 Methods

### 2.1 Study participants

This study utilized a combination of convenience sampling and snowball sampling methodologies. Participants were recruited from hospitals in China between August 2025 and January 2026. The inclusion criteria were as follows: (1) holding a valid professional qualification certificate; and (2) have at least 1 year of work experience, with at least 6 months of active nursing practice (including nursing management and clinical nursing work) in the current year. Exclusion criteria comprised a history of psychiatric disorders or cognitive impairment.

Regarding Latent Profile Analysis (LPA), the results indicate that when other design conditions are constrained, sample size does not exert a significant impact on statistical power (26–28). Specifically, sample size adjusted Bayesian information criteria (BIC) outperformed under the *N* = 100, 200 (28). The Bayesian Information Criterion (BIC) also exhibits excellent performance with small sample sizes [*N* = 200, 500; (29)]. Previous methodological studies suggest that a sample size of 300–500 is generally recommended as the minimum criterion for latent profile analysis (LPA), and that the reliability of results decreases when the sample size falls below 300 (30, 31). Accordingly, the present study adopted a minimum sample size of at least 300 participants.

### 2.2 Data collection

This was a single center cross sectional study. Between August 2025 and January 2026, a combined convenience and snowball sampling strategy was employed to recruit registered nurses from one tertiary general hospital located in Hangzhou, Zhejiang Province, China. This hospital was purposively selected based on the following criteria: (1) it is a tertiary comprehensive hospital with a bed capacity of 1,000 or more, ensuring a large and representative nursing workforce; (2) it has a well-established shift scheduling and nursing management system; and (3) it was accessible to the research team for on-site coordination, follow up, and quality assurance. Within the hospital, the nursing department distributed the survey link through internal official communication channels (department level WeChat groups) to all clinical nurses who met the eligibility criteria. All participants provided informed consent by clicking the agreement button on the first page of the online questionnaire. The introductory page provided participants with detailed information regarding the study objectives, methodology, and instructions for completion. Participants were informed that their involvement was voluntary and anonymous, and that they could withdraw at any time without any negative consequences. To ensure data integrity, each IP address was restricted to a single submission. The survey was presented in a sequential, page-by-page format and required approximately 8–10 min to complete. In addition, attention-check items were embedded in the survey to detect inattentive responding, and responses completed in less than 3 min were flagged for review.

### 2.3 Measurement

#### 2.3.1 Demographics

Demographic characteristics included gender, years of work experience, height, weight, monthly number of night shifts, and department. Participants' height and weight were collected via a self-administered questionnaire, and BMI was calculated from self-reported height and weight.

#### 2.3.2 Burnout

The job burnout of nurses in the cancer hospital was assessed using the Maslach Burnout Inventory-Human Services Survey (MBI-HSS). Originally developed by Maslach and Jackson (32), this instrument was validated by Feng et al. (33) and has demonstrated robust psychometric properties within the Chinese nursing population. The scale comprises 22 items categorized into three dimensions: Emotional Exhaustion (EE), Depersonalization (DP), and Personal Accomplishment (PA). Each item is rated on a 7-point Likert scale, ranging from 0 (“never”) to 6 (“every day”). Specifically, the Emotional Exhaustion dimension serves as the core component reflecting the individual stress level; Depersonalization captures the interpersonal context of burnout; and Personal Accomplishment represents the self-evaluation aspect (2). In the present study, Cronbach's alpha coefficient for the total scale was 0.827. Cronbach's alpha for the Emotional Exhaustion (EE), Depersonalization (DP) and Reduced Personal Accomplishment (PA) subscales was 0.932, 0.748 and 0.899.

#### 2.3.3 Emotional overeating and emotional undereating

Participants eating traits were assessed using the Adult Eating Behavior Questionnaire (AEBQ), originally developed by Hunot et al. (14) and validated in the Chinese population by He et al. (15) The AEBQ is a comprehensive instrument designed to measure both “food approach” and “food avoidance” traits. It consists of 35 items across eight dimensions: Hunger, Food Responsiveness, Emotional Overeating (EOE), Enjoyment of Food, Satiety Responsiveness, Emotional Undereating (EUE), Food Fussiness, and Slowness in Eating. Cronbach's alpha estimates of the eight subscales of the C-AEBQ ranged from 0.76 to 0.97, and the test–retest reliability coefficients of the subscales ranged from 0.50 to 0.77. Each item is rated on a 5-point Likert scale, ranging from 1 (“strongly disagree”) to 5 (“strongly agree”).

For the purpose of this study, we specifically focused on Emotional Overeating (EOE) and Emotional Undereating. We focused on EOE and EUE rather than binge eating because EOE&EUE captures a broader, non-clinical range of overeating in response to negative emotions, which is more appropriate for a general nurse population without diagnosed eating disorders. In the present sample, Cronbach's alpha for the EOE and EUE subscale was 0.951. In the latent profile analysis, both the Emotional Overeating (EOE) and Emotional Undereating (EUE) subscales of the AEBQ were entered as two separate indicator variables, rather than being combined into a single emotional eating composite. This decision allows the LPA to capture distinct patterns of appetitive response to negative emotions (i.e., overeating vs. under-eating) that may co-occur differently across burnout profiles.

### 2.4 Data analysis

Latent profile analysis (LPA) was performed using the three dimensions of the Maslach Burnout Inventory (emotional exhaustion, depersonalization, and reduced personal accomplishment) and the two dimensions of the Emotional Eating Scale (emotional overeating and emotional undereating) as profile indicators. A hybrid LPA approach was adopted to identify distinct subgroups characterized by joint patterns of burnout and emotional eating. Enumeration began with an initial single-profile model, and solutions containing one to five profiles were sequentially extracted and compared. Model fit was evaluated using the Akaike Information Criterion (AIC), Bayesian Information Criterion (BIC), and sample-size adjusted BIC (aBIC), with lower values indicating a superior model fit (34, 35). Classification accuracy was assessed via entropy (ranging from 0 to 1), where values closer to 1 denote more precise individual classification. Additionally, the Lo–Mendell–Rubin adjusted likelihood ratio test (LMR), and the bootstrap likelihood ratio test (BLRT) were employed to compare neighboring models (34); a significant *p*-value indicates that the k-class model provides a significantly better fit than the k−1 class model (35).

In this study, the number of latent profiles was selected by comprehensively considering the ratio and interpretability of each profile together with the information index and entropy value (36).

### 2.5 Ethical considerations

The research was conducted with prior approval by the Institutional Review Board at the institution where the author is affiliated [Approval No. (2025B) IIT Ethics 1117], about the purpose of the research, research method, information protection, risk factors, etc. All participants were informed about the research at the beginning of the online survey and voluntarily provided consent by agreeing to the online survey system. The online survey procedure, data storage, and management were conducted with precautions reported and confirmed by the IRB at the institution where the first author is affiliated.

## 3 Results

### 3.1 Demographic and professional characteristics of the nursing team

**Figure 1:**
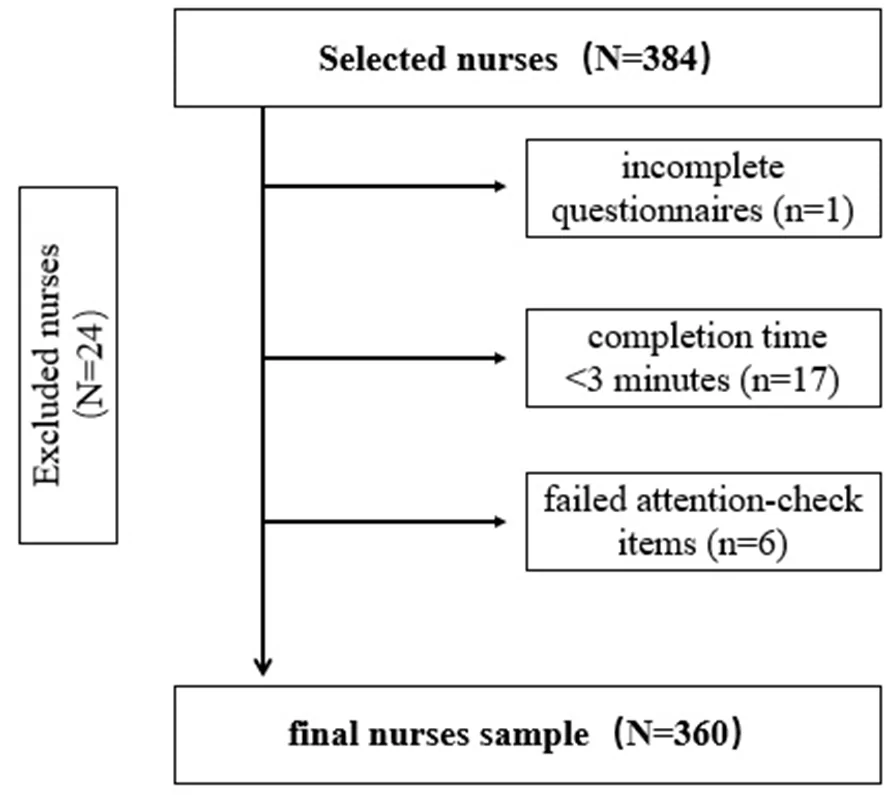
Flowchart of the sampling process of nursing home residents.

From the 384 potential residents, 24 did not meet the inclusion criteria for the study (Figure 1. Flowchart of the sampling process of nurses). A total of 360 nursing professionals with no missing data were included in this study (Table 1). The mean age of the participants was 29.6 years (SD = 4.6), ranging from 22 to 54 years. The sample was predominantly female (91.4%) and primarily deployed in Internal Medicine (39.4%) and Surgery (31.1%). In terms of professional tenure and workload, 41.7% of the participants possessed more than 10 years of experience, and the majority (83.9%) performed 8 or fewer night shifts per month. Descriptive statistics showed that the mean Body Mass Index (BMI) of the sampled nurses was 22.1 ± 3.52 kg/m^2^ (range: 12.1–40.6).

**Table 1:** Socio-demographic and work-related characteristics of participants (*N* = 360).

Variable	Category	*n*	%	Cumulative %
Gender	Female	329	91.4%	91.4%
	Male	31	8.6%	100.0%
Years of work	1–2 years	89	24.7%	24.7%
	3–5 years	42	11.7%	36.4%
	6–10 years	79	21.9%	58.3%
	>10 years	150	41.7%	100.0%
Department	Internal medicine	142	39.4%	39.4%
	Surgery	112	31.1%	70.6%
	Outpatient department	63	17.5%	88.1%
	ICU	27	7.5%	95.6%
	Emergency	9	2.5%	98.1%
	Operating room	7	1.9%	100.0%
Monthly night shifts	0–4 times	155	43.1%	43.1%
	5–8 times	147	40.8%	83.9%
	9–12 times	54	15.0%	98.9%
	>12 times	4	1.1%	100.0%

### 3.2 Latent profile models

**Figure 2:**
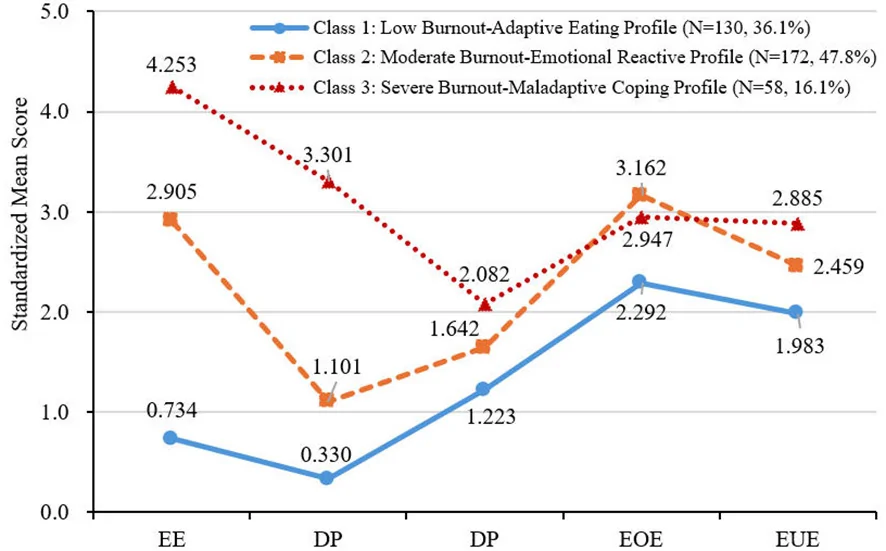
Latent profile analysis (LPA): 3-class solution trends.

As specified in the Methods, the LPA included the three burnout dimensions (with PA reverse-coded) and the two independent emotional eating dimensions (EOE and EUE) as indicator variables. The fit indices for the latent profile analysis are summarized in Table 2. The selection of the optimal model was justified based on three criteria: (1) the AIC, BIC, and SSABIC values decreased as the number of profiles increased and began to plateau at the three-profile model, as further partitioning yielded diminishing returns in descriptive fit; (2) an entropy value of 0.810 (greater than 0.80) indicated high classification distinctiveness, with the average latent class probabilities ranging from 0.899 to 0.928, which demonstrated a classification accuracy of approximately 90%; and (3) the LMR-LRT (*p* = 0.0003) and BLRT (*p* < 0.001) values were statistically significant, confirming a significant improvement in model fit over the two-profile solution. Although the four- and five-class models continued to show improvements in log-likelihood values, with LMR and BLRT tests remaining significant, the final decision was to reject these more complex solutions for the following reasons. First, both the four- and five-class models yielded latent classes with extremely small sample sizes (4.3% and 4.1%, respectively, corresponding to *n* = 15), falling below the conventional 5% threshold (37); such small classes lack statistical stability and are theoretically difficult to interpret as distinct population subgroups. Second, the decrement in BIC values exhibited a clearly diminishing marginal effect as the number of classes increased (particularly, the decline from the four- to five-class model was only approximately 15 points). Third, the five-class model showed a decline in classification accuracy (Entropy). Therefore, guided by the principle of parsimony and the clinical interpretability of each class, the four- and five-class models were excluded, and the three-profile model was retained as the final solution. Table 3 and Figure 2 present the distribution of burnout and eating-related psychological characteristics among nurses in the three-class model. The discriminant validity of the derived latent profiles was assessed via ANOVA, comparing the three classes on each of the five indicator variables. Results (Table 3) revealed significant between-profile differences across all dimensions (all *p* < 0.001), with partial eta-squared values ranging from 0.051 to 0.734. Of particular note, the core burnout facets—emotional exhaustion (ηp^2^ = 0.734) and depersonalization (ηp^2^ = 0.725)—showed markedly large effects, suggesting robust discriminative power among the classes. Concurrently, the emotional eating indicators also yielded moderate to large effect sizes (EOE: ηp^2^ = 0.153; EUE: ηp^2^ = 0.110), lending further support to a stable typological relationship between progressive burnout stages and distinct maladaptive eating behaviors. Class 1 constituted the second largest subtype (*n* = 130, 36.111%), exhibiting the lowest levels of burnout and eating-related psychological symptoms. Class 2 was the largest subtype (*n* = 172, 47.778%), characterized by moderate levels of burnout but the highest scores on the emotional eating dimension. Class 3 was the smallest subtype (*n* = 58, 16.111%), showing the highest levels of burnout and external eating psychological distress. As illustrated in Figure 2, although these classes differed in their overall distributions, their response patterns revealed distinct psycho-behavioral differentiation features (e.g., Class 2 showed a marked spike in emotional eating scores, even exceeding those of the severe burnout group; whereas Class 3 scored substantially higher than the other subtypes on the dimensions of emotional exhaustion and depersonalization). Based on the results, Class 1 was labeled the “Low Burnout-Adaptive Eating” subtype, Class 2 the “Moderate Burnout-Emotional Reactive” subtype, and Class 3 the “Severe Burnout-Maladaptive Coping” subtype.

**Table 2:** Fit indices for latent profile models of burnout among nurses (*N* = 360).

K	FP	LL	AIC	BIC	SSABIC	Entropy	LMR-LRT (p)	BLRT (p)	Proportions
1	10	−2,929.755	5,879.509	5,918.370	5,886.645	—	—	—	100.0%
2	16	−2,803.991	5,639.982	5,702.160	5,651.400	0.865	0.0000	0.0000	76.9%/23.1%
3	22	−2,731.794	5,507.588	5,593.082	5,523.287	0.810	0.0003	0.0000	36.8%/46.9%/16.2%
4	28	−2,667.703	5,391.406	5,500.217	5,411.387	0.853	0.0000	0.0000	45.8%/4.3%/33.7%/16.2%
5	34	−2,642.563	5,353.125	5,485.253	5,377.387	0.818	0.0081	0.0000	4.1%/11.2%/29.4%/40.1%/15.1%
6	40	−2,622.504	5,325.009	5,480.453	5,353.552	0.798	0.4448	0.0000	11.0%/20.9%/23.0%/11.9%/4.0%/29.2%

**Table 3:** Means and standard errors of burnout and eating behavior dimensions across three latent profiles.

Variables	Class 1: low burnout-adaptive eating (*n* = 130)	Class 2: moderate burnout-emotional reactive (*n* = 172)	Class 3: severe burnout-maladaptive coping (*n* = 58)	F (df = 2,357)	*p*	ηp^2^
EE	0.73	2.91	4.25	492.00	<0.001	0.734
DP	0.33	1.10	3.30	470.00	<0.001	0.725
PA	1.22	1.64	2.08	9.67	<0.001	0.051
EOE	2.29	3.16	2.95	32.20	<0.001	0.153
EUE	1.98	2.46	2.89	22.00	<0.001	0.110

### 3.3 Profile differences in demographic and work-related characteristics

The Kruskal-Wallis test and the Dwass-Steel-Critchlow-Fligner (DSCF) *post-hoc* pairwise comparison were employed to examine differences in demographic and work-related ordinal variables across the identified profiles. Concurrently, because Body Mass Index (BMI) exhibited heterogeneity of variance, Welch's analysis of variance (ANOVA) was utilized for inter-group comparisons. Detailed results regarding the comparisons of these variables across the distinct profiles are presented in Table 4.

**Table 4:** Comparison of demographic and work-related ordinal variables across the three latent profiles (*N* = 360).

Variable	Test statistic	df	*p*	Effect size (ε^2^)	*Post-hoc* pairwise comparisons (DSCF)
Years of work experience	*χ^2^* = 12.220	2	0.002	0.034	Class 3 <Class 2 (*W* = −4.98, *p* = 0.001); Class 3 vs. Class 1 (*W* = −3.25, *p* = 0.056); Class 1 vs. Class 2 (*p* = 0.377)
Monthly night shift frequency	*χ^2^* = 6.186	2	0.045	0.017	Class 3 > Class 1 (*W* = 3.49, *p* = 0.036); Class 2 vs. Class 3 (*p* = 0.197); Class 1 vs. Class 2 (*p* = 0.488)
Department	*χ^2^* = 0.252	2	0.882	0.001	All pairwise comparisons *ns* (*p* > 0.85)
BMI (kg/m^2^)	*F* = 2.60 (Welch's)	2, 229	0.077	—	Not applicable (overall *p* > 0.05)

Class 1 = Low Burnout-Adaptive Eating (*n* = 130); Class 2 = Moderate Burnout-Emotional Reactive (*n* = 172); Class 3 = Severe Burnout-Maladaptive Coping (*n* = 58).

For BMI, Welch's F test is reported due to violation of homogeneity of variance; degrees of freedom are (df1, df2).

The Kruskal-Wallis test results revealed statistically significant differences across the three profiles in terms of years of work experience (χ*2* = 12.220, df = 2, *p* = 0.002, ε*2* = 0.034) and monthly night shift frequency tiers (χ*2* = 6.186, df = 2, *p* = 0.045, ε*2* = 0.017). In the *post-hoc* pairwise comparisons for work experience, the tenure of nurses in the “Severe Burnout-Maladaptive Coping” profile (Class 3) was significantly shorter than that of their counterparts in the “Moderate Burnout-Emotional Reactive” profile (Class 2) (*W* = −4.98, *p* = 0.001); however, its difference from the “Low Burnout-Adaptive Eating” profile (Class 1) did not reach statistical significance (*W* = −3.25, *p* = 0.056). No statistically significant difference was observed between Class 1 and Class 2 (*p* = 0.377). Regarding the monthly night shift frequency, the night shift tiers in the “Severe Burnout-Maladaptive Coping” profile (Class 3) were significantly higher than those in the “Low Burnout-Adaptive Eating” profile (Class 1) (*W* = 3.49, *p* = 0.036), whereas the remaining pairwise comparisons yielded no statistically significant differences (Class 1 vs. Class 2, *p* = 0.488; Class 2 vs. Class 3, *p* = 0.197).

Furthermore, the distribution across departments did not differ significantly among the three profiles (χ*2* = 0.252, df = 2, *p* = 0.882, ε*2* = 0.001), indicating that the composition of nurses from different clinical departments was largely homogeneous within each profile. For BMI, Welch's ANOVA showed that the mean differences among the three groups were not statistically significant (*F* = 2.60, df1 = 2, df2 = 229, *p* = 0.077), suggesting that the current body mass index levels of the nurses across all profiles were comparable.

### 3.4 Multinomial logistic regression analysis of latent profile for nurse burnout and emotional eating

To evaluate the concurrent predictive capacity of operational stressors and biographical tenure on profile membership, a multinomial logistic regression was executed via the automatic Mplus 3-step (R3STEP) (Muthén & Muthén; Los Angeles, CA, USA) procedure, with both monthly night shifts and years of experience treated as ordinal predictors (each coded from 1 to 4). As detailed in Table 5, after adjusting for measurement error, night shift frequency and professional experience exhibited significant, counteractive step-wise effects on profile assignment, whereas Body Mass Index (BMI) retained no statistical significance across all axes of comparison (all *p* > 0.70).

**Table 5:** Multinomial logistic regression predicting profile membership.

Class comparisons	BMI			Night shifts/month ^a^			EXPERIENCE ^b^		
Class Comparisons	β	*p*	OR (95% CI)	β	*p*	OR (95% CI)	β	*p*	OR (95% CI)
Class 2 vs. Class 1	−0.013	0.757	0.987 (0.91, 1.07)	0.216	0.294	1.241 (0.83, 1.86)	0.201	0.091	1.222 (0.97, 1.54)
Class 3 vs. Class 1	−0.016	0.712	0.984 (0.90, 1.07)	0.546	0.014^*^	1.726 (1.12, 2.67)	−0.332	0.026^*^	0.718 (0.54, 0.96)
Class 3 vs. Class 2	−0.003	0.944	0.997 (0.92, 1.08)	0.330	0.124	1.391 (0.91, 2.12)	−0.533	0.001^***^	0.587 (0.43, 0.79)

β represents the log-odds regression coefficient; OR represents the odds ratio; CI = Confidence Interval. ^*^p < 0.05, ^***^p < 0.001.

^a^ Monthly night shift levels are ordinally coded as: 1 = 0–4 shifts; 2 = 5–8 shifts; 3 = 9–12 shifts; 4 = > 12 shifts.

^b^ Professional tenure levels are ordinally coded as: 1 = 1–2 years; 2 = 3–5 years; 3 = 6–10 years; 4 = > 10 years.

Specifically, with the healthy “Low Burnout-Adaptive Eating Profile (Class 1)” established as the baseline horizontal reference: the monthly night shift classification acted as a step-wise risk multiplier, wherein each one-level increase in the night shift category (e.g., from 0–4 shifts to 5–8 shifts) significantly escalated the risk of individuals transitioning into the “Severe Burnout-Maladaptive Coping Profile (Class 3)” relative to remaining in Class 1 by 72.6% (β = 0.546, S.E. = 0.222, *p* = 0.014, OR = 1.726). Concurrently, professional seniority provided incremental defense; each one-level advancement in the experience hierarchy significantly reduced the log-odds of being classified into Class 3 relative to Class 1 by 28.2% (β = −0.332, S.E. = 0.149, *p* = 0.026, OR = 0.718). Furthermore, when re-parameterized with the “Moderate Burnout-Emotional Reactive Profile (Class 2)” serving as the referent group, the defensive insulation of the experience ranks intensified, with each step-wise progression in seniority significantly lessening the odds of escalating from the moderate into the severe dysfunction profile (Class 3) by 41.3% (β = −0.533, S.E. = 0.154, *p* = 0.001, OR = 0.587).

## 4 Discussion

Currently, nurses worldwide continue to experience varying degrees of burnout (38–40), which seriously affects their physical and mental health and the long-term healthy development of healthcare systems. The empirical findings derived from this investigation provide person-centered validation for the intrinsic linkages between occupational stressors and continuous disordered eating behaviors among clinical nurses, while substantively advancing the predictive taxonomy and causal resolution of prior literature. Traditional variable-centered inquiries (41, 42) have widely documented that elevated job stress significantly correlates with individuals' susceptibility to disordered eating expressions, conceptualizing abnormal appetitive traits predominantly as a passive coping mechanism to buffer chronic workplace distress and negative affective states. The finding that eating behavior serves as a maladaptive compensatory mechanism in the context of burnout among nurses is consistent with evidence from a Thai sample (19).

Consistent with previous findings (43), this study reveals that high-frequency night shift/rotating shift work does not function as an isolated occupational arrangement but rather serves as one of the contributing factors that trigger a cascade of psychological resource depletion and maladaptive eating behaviors among nurses. Moreover, the adverse effects of night shifts are particularly pronounced among junior nurses, necessitating managerial strategies that ensure a reasonable allocation of night shift duties to prevent this population from being prematurely pushed into a vicious cycle of resource exhaustion during their critical career adaptation phase. Future research should further employ Ecological Momentary Assessment (EMA) to capture the dynamic real-time associations between eating behaviors and emotional fluctuations before and after night shifts, thereby precisely identifying the optimal time window for targeted interventions.

Furthermore, the results of this study revealed a distinctive nonlinear pattern, in which the highest score for emotional overeating (EOE) did not occur in the Severe Burnout–Maladaptive Coping group (Class 3), but rather in the Moderate Burnout–Emotional Reactive group (Class 3). Although nurses in the moderate burnout group were exposed to occupational stress, they remained relatively high emotional sensitivity and exhibited pronounced acute emotional reactions to heavy clinical workloads and frequent night shifts. Consequently, they frequently resorted to overeating as an immediate regulatory strategy to seek hedonic comfort, which resulted in their peak EOE scores. In contrast, nurses in the Severe Burnout–Maladaptive Coping group (Class 3) scored at extreme levels on both emotional exhaustion and depersonalization. This extremely high level of depersonalization essentially reflects a profound blunting of the perception of internal negative emotions and the emergence of behavioral detachment, as a result of long-term, unbearable stress (32). This nonlinear pattern suggests to nursing managers that a decline in overeating scores should by no means be interpreted as an improvement in dietary behavior; rather, it may signal a perilous progression of psychological defense mechanisms toward profound emotional disengagement and unresponsiveness. Therefore, hospitals should regard the moderate burnout group, which accounts for 47.8% of the sample, as the “golden window period” for precise intervention. Early detection and intervention for this subgroup are crucial, and management efforts should focus on expanding channels for emotional catharsis and providing immediate alternative soothing resources. Such precision-guided organizational support holds profound promise for arresting early-career loss spirals, stabilizing workforce retention, and preserving clinical patient safety benchmarks across the healthcare ecosystem.

## 5 Limitations

Several limitations of this study should be acknowledged. First, the combination of convenience and snowball sampling, together with a predominantly female sample (91.4%) drawn mainly from internal medicine and surgical departments, may limit generalizability to male nurses, primary care settings, or other specialty populations. Second, the cross-sectional design precludes causal inferences regarding the relationships among burnout, emotional eating, night-shift frequency, and work experience; longitudinal studies are needed to examine dynamic trajectories consistent with resource conservation theory. Third, reliance on self-reported data may introduce social desirability, recall, and common method bias, despite the inclusion of attention-check items. Fourth, the study focused exclusively on emotional overeating and undereating without examining other eating patterns (e.g., food addiction, restrained eating) or incorporating objective measures such as dietary records or physiological indicators (e.g., cortisol). Fifth, although the sample size (*N* = 360) met the minimum requirements for LPA, the relatively small severe burnout subgroup (*n* = 58) may affect the stability of parameter estimates. Additionally, the study did not control for potential confounders such as social support, shift-work adaptability, or prior psychological treatment history, nor did it exclude individuals with confirmed eating disorders (beyond psychiatric history). BMI was based on self-report rather than standardized measurements, which may introduce bias and attenuate its association with outcomes; null findings regarding BMI should therefore be interpreted cautiously, and future studies should employ standardized anthropometric protocols to clarify its potential role. Last, the sample was recruited from southeastern China, which may limit generalizability to other regions given marked inter-regional disparities in healthcare resources, workload, and socioeconomic conditions; multi-center studies covering eastern, central, and western China are needed to validate our findings.

Additionally, our multinomial logistic regression models omitted several critical confounders, including age, department, social support, and work environment characteristics. This omission may introduce bias; for example, the observed protective effect of professional experience might be partially confounded by age-related emotional maturation, while stress exposure significantly varies by department. Furthermore, from a COR theory perspective, social support and favorable work environments serve as vital external resources that buffer occupational stress. Failing to control these variables may inflate the independent effects of night shifts and tenure. Future research should integrate these socio-ecological factors into more comprehensive models to isolate their specific pathways.

## 6 Conclusion

Using latent profile analysis, this study identified three distinct profiles among clinical nurses: “Low Burnout–Adaptive Eating,” “Moderate Burnout–Emotional Reactive,” and “Severe Burnout–Maladaptive Coping.” Logistic regression analyses revealed that monthly night-shift frequency was significantly associated with an increased likelihood of classification into the more severe burnout profiles, whereas longer professional experience appeared to confer a protective effect; body mass index, however, showed no significant association with profile membership. These findings are consistent with the Conservation of Resources (COR) theory, in that recurrent night-shift work may precipitate the depletion of self-regulatory resources, which in turn may predispose individuals to emotional eating as a maladaptive compensatory strategy. Nevertheless, given the cross-sectional nature of the study design, causal relationships among night-shift exposure, burnout, and emotional eating cannot be established. Due to the cross-sectional, single-center design of this study, definitive causal relationships among job burnout, emotional eating, night-shift frequency, and work experience cannot be inferred. Future research should incorporate longitudinal follow-up designs to investigate the dynamic trajectories of individual resource conservation and depletion over time. From a practical standpoint, these findings underscore the need for targeted organizational interventions: optimizing shift scheduling to reduce monthly night-shift frequency, and implementing resource-based programs—such as mindful eating or emotion-regulation training—particularly for less experienced nurses with high shift loads, so as to mitigate early-career resource loss, enhance workforce retention, and safeguard patient safety.

## Data Availability

The original contributions presented in the study are included in the article/supplementary material, further inquiries can be directed to the corresponding author/s.
